# The associations between behavioral-psychological symptoms of dementia (BPSD) and coping strategy, burden of care and personality style among low-income caregivers of patients with dementia

**DOI:** 10.1186/s12889-019-6868-0

**Published:** 2019-06-13

**Authors:** Aishah Diyana Baharudin, Normah Che Din, Ponnusamy Subramaniam, Rosdinom Razali

**Affiliations:** Psychology Programme, Faculty of Health Sciences, Universiti Kebangsaan Malaysia, Jalan Raja Muda Abdul Aziz, 50300 Kuala Lumpur, Wilayah Persekutuan Malaysia

**Keywords:** Behavioral-psychological symptoms of dementia, Caregiver burden, Coping strategies, Personality style, Dementia, Mediation

## Abstract

**Background:**

The main aim of this study was to determine the association between Behavioral and Psychological Symptoms of Dementia (BPSD) and caregiver burden, and the mediating role of coping strategy and personality style of caregivers to patients with dementia (PWD).

**Methods:**

This cross-sectional study was conducted among 202 caregivers to PWD in home-based settings. Recruited caregivers were administered questionnaires regarding BPSD which was measured using *Neuropsychiatric Inventory-Questionnaire* (NPI-Q), caregiver burden using *Zarit Burden Interview* (ZBI), *Brief COPE for* coping strategies and *Big-Five Inventory* which measured personality traits.

**Results:**

Majority of the caregivers were female (71.3%), aged 50 and above (55%), single (46%), married (43.6%), working full time (45%) while the rest work part time (22.3%), unemployed (7.4%) and retiree (25.2%), and majority were parents (58.9%) and spouse (18.3%). The duration of caregiving was less than a year (33.7%) while the rest are more than a year. Results demonstrated that the most frequent types of BPSD exhibited by PWD was irritability, followed by apathy and agitation. All of the types of BPSD showed to be significantly correlated to caregiver burden except for anxiety, elation and appetite. Of personality traits, only conscientiousness was found to mediate the relationship between BPSD and caregiver burden (*p* < .05). Self-distraction, active coping, planning and acceptance were the coping strategies that demonstrated to have mediation effect on the relationship between BPSD and caregiver burden.

**Conclusion:**

Presentation of BPSD is correlated to caregiver burden which is partially mediated by coping strategies and personality styles.

## Background

Dementia is a neurodegenerative disorder that is progressive in nature involving the impairment of multiple higher cortical functions [1]. Symptoms of dementia comprise of two major groups which can be divided into cognitive symptoms and non-cognitive symptoms. Cognitive symptoms focus on impairment of memory, especially on learning of new material and short-term memory which is a key early symptom. Non-cognitive symptoms constitute of neuropsychiatric symptoms, also known as behavioral-psychological symptoms of dementia (BPSD). Behavioral symptoms are often identified based on observation of patients with dementia (PWD) such as physical aggression, screaming, restlessness, agitation, wandering, culturally inappropriate behaviors, sexual disinhibition, hoarding, cursing and shadowing [2]. Psychological symptoms are usually evaluated based on interviews with patients and informants which includes anxiety, depressive mood, hallucinations and delusions [3]. The emergence of BPSD can occur during any stage in dementia where patients demonstrate at least one type of BPSD [4]. In Austria, the highest prevalence of BPSD was disruptive behaviors such as agitation and aggression [5] while in Asia, sleep disturbance, irritability and apathy were found to be the most common BPSD among patients of Alzheimer’s in China [6]. In Malaysia, apathy was reported to be the most prevalent with 83.2%, followed by agitation (60%) and sleep disturbance (53.8%) [7]. These inconsistent findings on BPSD could be due to the differences in methodology such as settings, designs and instruments. However, despite these differences, the epidemiology of BPSD seemed clear and consistent [8].

Behavioral issues have challenged the caregivers of PWD with an increase rate of morbidity and mortality for both the care recipient and caregiver. PWD with BPSD such as over activity and aggression are likely to be susceptible to abuse and neglect whereby these behavioral symptoms and the effects of challenging behavior can cause great frustration for the caregivers [9]. Progression of dementia reflects the severity of impairment on cognitive and non-cognitive functioning, hence influencing the caregivers’ focus of care. It was suggested that differentiated care needs to be given during early and middle stages of dementia due to emergence of behavioral symptoms that are reported as challenging by the caregivers [10]. Dementia caregivers have shown stress and burnout with high prevalence of clinical depression and anxiety [11] as they are dealing with cognitive deterioration as well as challenging behavior. More than 80% of Alzheimer’s disease caregivers frequently report high levels of stress and half report suffering from depression and anxiety [12–17]. BPSD has been estimated to affect 90% of PWD over the course of the illness and it has resulted in negative consequences such as distress in caregiver and patients, long term hospitalization, misuse of medication and increased health care costs. BPSD can also result in premature institutionalization, increased costs of care, and significant loss of quality of life for patients and their families and caregivers [18].

The caregiver burden is influenced based on many psychosocial factors such as kinship, social environment and culture [15]. In Malaysia, caregiver burden was found to be significantly associated with both ethnicity and informal support [19]. Multiple studies have focused on impact of behavioral disturbance on caregiver burden. In the caregiving role, caregivers may be confronted with numerous, often unpredictable, stressors associated with their responsibilities. Previous research has shown that coping strategies among caregivers are related to outcomes such as depression and life satisfaction [20, 21]. Caregiver distress has been found to increase with the use of emotion-focused coping strategies [22]. Caregivers who reported less use of problem-focused coping and greater use of emotion-focused coping also reported experiencing more burnout [23] and reported fewer depressive symptoms [24]. Personal resource such as personality of caregivers has been identified as potentially important factor which has not received much attention [25]. There is indeed increasing evidence that individual differences in personality may affect how caregivers experience and respond to the caregiving role [26]. Based on the context of the five-factor taxonomy of personality, neuroticism is the most extensively studied personality trait in dementia caregiving research [26]. Hooker et al. [27] revealed that personality was related to mental and physical health outcomes among spouse caregivers of PWD. Past research has investigated why caregivers under similar circumstances exhibit variability in their ability to adapt to caregiving stressors. Diathesis-Stress Model of Psychopathology posits that variability in outcomes for caregivers experiencing similar stressors is related to underlying personality dispositions making them more vulnerable towards negative outcomes [28]. High levels of neuroticism in dementia carers predict higher perceived stress [29], limited access to social support [30], worse physical heath [31], and higher depressive symptoms [32]. In contrast, caregivers who score high in extraversion and agreeableness experience lower depressive symptoms and burden [33].

In Malaysia, research on health status and well-being of older adults using multidimensional approaches has been rapidly growing [34–38]. However, research on BPSD in PWD is still lacking. Despite intensive study on direct relationship between BPSD and caregiver burden, research exploring the indirect effects of personality styles and coping styles has remained limited. The aim of the current research was to investigate the associations between BPSD and caregiver burden with coping strategy and personality style of caregivers as the mediation.

## Method

### Research design

A cross-sectional research design was used in the current study.

### Setting and participants

The primary scope of this study comprised of caregivers to patients who are registered under Alzheimer’s Disease Foundation Malaysia (ADFM) centre in Petaling Jaya, Malaysia.

There were 202 caregivers who volunteered to join the study. They attended the weekly or monthly activities. Sample size was calculated using multivariate statistics which adhered to the sample size calculation of multiple regression analysis by Green [39] where *N* > 50 + 8 m in which N represented number of participants and m represented number of independent variables (i.e. Brief COPE with 14 independent variables and Big Five Inventory with 5 independent variables) thus, in total of 19 independent variables. Participants were recruited through purposive sampling who provided written consent to participate in the study. Following that, they were randomly selected from the list provided by the ADFM to proceed with parametric statistical analysis.

The inclusion criteria of caregivers included that they must be able to read and understand English and Malay language and aged 18 and above. The PWD of these caregivers must be registered under the association of ADFM and were previously diagnosed with mild to severe stages of dementia by a specialist working in a hospital. Caregivers comprised of family caregivers as well as formal caretakers such as housemaids or personal helper as long as caregiving is provided within vicinity of home-based setting.

Exclusion criteria of caregivers were those diagnosed to have chronic medical or psychiatric illness and neurological conditions as well as caregivers to patients from residential facilities and institution. Patients with other cognitive impairment (e.g. Traumatic Brain Injury, Mild Cognitive Impairment, Amnesia or other secondary organic causes of memory loss that are not primarily associated to dementia) were also excluded from the study.

### Materials

A self-administered questionnaires consisting of four main components was used: a) Behavioral and Psychological Symptoms of Dementia (BPSD) which was measured using the Neuropsychiatric-Inventory Questionnaire (NPI-Q); b) burden as a caregiver was measured using Zarit Burden Interview (ZBI), c) coping strategies was measured using the Brief COPE; and lastly, d) personality style was measured using Big-Five Inventory (BFI).

The NPI-Q [40] measures dementia-related behavioral symptoms including subdomains on delusions, hallucinations, agitation/aggression, depression/dysphoria, anxiety, elation, apathy, disinhibition, irritability, motor disturbance, night-bedtime behaviors and appetite. The Zarit Burden Interview (ZBI) [41] is a self-report questionnaire comprising of 22 questions to measure subjective burden among caregivers of adults with dementia. The ZBI was adapted to several languages, and the internal consistency ranged from .85 to .94. The validated Malay version of ZBI demonstrated good psychometric properties with an internal consistency of .89 in assessing the caregiver burden among local Malaysian population [42]. The 28-item self-reported Brief COPE scale assesses a broad range of coping responses among adults [43]. The higher score represents greater coping strategies used by the respondents. In total, 14 dimensions were covered by this scale which comprised of self-distraction, active coping, denial, substance use, use of emotional support, use of instrumental support, behavioral disengagement, venting, positive reframing, planning, humor, acceptance, religion and self-blame. The instrument acquired Cronbach’s alphas ranged from .88 to .81 [43]. The Malay Version of Brief COPE Scale is a reliable and valid instrument which could be applied for the Malaysian population, with regards on its acceptable internal consistency. Cronbach’s alpha value of the Malay version Brief COPE was .83 [44]. The personality test Big-Five Inventory (BFI) [45] provides a score for each of the Big Five personality traits (Conscientiousness, Agreeableness, Emotional Stability, Extroversion and Intellect or Openness). BFI has also been validated in the Malaysian context whereby the reliability of the measures has been found consistent and indicates the homogeneity of the items in the measures [46].

### Statistical analysis

SPSS IBM Version 23 was used to analyze the data using descriptive and inferential statistics. BPSD was analyzed using frequency and percentage. The association between BPSD and caregiver burden was analyzed using bivariate correlation. Multiple Hierarchical Regression and Multiple Mediation (INDIRECT) were used to examine the mediating role of coping strategies and personality styles between behavioral-psychological symptoms of dementia and caregiver burden. The data was found to be normally distributed and met the assumptions for multiple regression allowing for parametric analysis. Since the subjects were randomly selected from the list provided by ADFM, multiple regression analysis can be conducted.

**Fig. 1:**
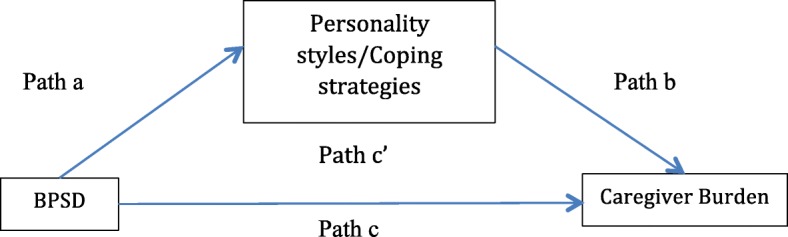
Hypothesized mediation effects of personality or coping strategies between BPSD and caregiver burden

To examine the mediating role of proposed variables, Baron and Kenny [47] method was used to analyse the mediation hypotheses. In this method, the mediation effect is identified when the predictors (BPSD) significantly associate with both the mediator (personality styles or coping strategies) (path a) and outcome (path c) and independent variable and mediator predicting the dependent variable (path c’) which must be fulfilled in the results to support mediation. Figure 1 showed the hypothesized mediation effects of personality or coping strategies between BPSD and caregiver burden.

There will be four steps involved in conducting the mediation analysis. Steps 1 to 3 is conducted to establish that zero-order relationships among the variables. If one or more of these relationships are nonsignificant, researchers usually conclude that mediation is not possible or likely. Assuming there are significant relationships from Steps 1 through 3, analysis proceeds to Step 4. In the Step 4 model, some form of mediation is supported if the effect of M (variable that mediates the effects of X on Y, path b) remains significant after controlling for X (cause/predictor). Y is the outcome of the study. If X is no longer significant when M is controlled, the finding supports full mediation. If X is still significant (i.e., both X and M both significantly predict Y), the finding supports partial mediation [48]. Complete mediation is present when the independent variable (X) no longer influences the dependent variable (Y) after the mediator has been controlled and all of the above conditions are met. Partial mediation occurs when the independent variable’s influence on the dependent variable is reduced after the mediator is controlled.

## Results

### Demographic characteristics of participants

Measures of frequency and percentage for participants according to gender, race, age, marital status and occupational status are displayed in Table 1 below. Majority of recruited caregivers were females (71.3%); age 50 years and above (55%); single (46.0%), married (43.6%); 45% work full time while the rest work part-time (22.3%), unemployed (7.4%) and retiree (25.2%). Majority of the caregivers are parents (58.9%) with the duration of caregiving less than a year (33.7%) while the rest have taken care of the PWD more than a year.

**Table 1:** Sociodemographic characteristics of participants (Caregivers, *n* = 202)

Characteristics	Frequency (*n* = 202)	Percentage
Gender		
Male	58	28.7
Female	144	71.3
Age		
18–20	13	6.4
21–30	31	15.3
31–40	21	10.4
41–50	26	12.9
50 and above	111	55.0
Marital Status		
Single	93	46.0
Married	88	43.6
Divorced	15	7.4
Others	6	3.0
Occupational status		
Working full time	91	45.0
Working part-time	45	22.3
Unemployed	15	7.4
Retiree	51	25.2
Relationship of caregivers to PWD		
Spouse	37	18.3
Parent	119	58.9
Sibling	20	9.9
Family relatives	10	5.0
Employer	16	7.9
Duration of caregiving		
Less than 1 year	68	33.7
1 year – 3 years	65	32.3
3 years – 5 years	14	6.9
5 years – 7 years	32	15.8
7 years and above	23	11.4

Measures of frequency and percentage for reports on frequency of BPSD among people with dementia as reported by their respective caregivers (*N* = 202) are displayed in Table 2. Irritability was the most frequently reported type of BPSD (84.2%) followed by apathy (80.7%) and agitation (77.2%) as indicated by caregivers. The least reported types of BPSD were elation (32.7%) followed by motor disturbance (57.5%) and appetite (59.4%).

**Table 2:** Frequency of BPSD among PWD reported by caregivers

Types of BPSD	Frequency	Percentage
Delusions	143	70.8
Hallucinations	127	75.0
Agitation/Aggression	156	77.2
Depression/Dysphoria	133	65.8
Anxiety	134	66.3
Elation/Euphoria	66	32.7
Apathy	163	80.7
Disinhibition	124	61.4
Irritability	170	84.2
Motor Disturbance	116	57.5
Nighttime Behaviours	144	71.3
Appetite	120	59.4

### Correlation between types of BPSD, personality, coping strategies and total caregiver burden

Table 3 indicates majority of BPSD types have significant positive correlation with caregiver burden (i.e. delusion, agitation, irritability and nighttime behavior, hallucination, depression, apathy, disinhibition and motor disturbance). However, Anxiety, elation and appetite are not significantly correlated to Total Caregiver Burden.

**Table 3:** Bivariate correlation of types of BPSD and total caregiver burden

	Total Caregiver Burden
Types of BPSD	
Delusion	.121*
Hallucination	.184**
Agitation	.115*
Depression	.157**
Anxiety	.048
Elation	.059
Apathy	.379**
Disinhibition	.201**
Irritability	.126*
Motor Disturbance	.144**
Night time Behaviour	.113*
Appetite	.079
Total BPSD	.199**
Personality	
Extroversion	−.186**
Agreeableness	−.342**
Conscientiousness	−.391**
Neuroticism	.282**
Openness	.045
Coping strategies	
Self-Distraction	.478**
Active Coping	.325**
Denial	.156*
Substance Use	.056
Emotion support	.053
Behaviour Disengagement	−.079
Instrumental Support	.235**
Venting	.358**
Positive Reframing	.255**
Planning	.393**
Humour	−.043
Acceptance	.427**
Religion	.024
Self-Blame	−.017

*Note*. * *p* < .05 level (1-tailed)

** *p* < .01 level (1-tailed)

Majority of personality traits of the caregivers have significant negative correlation with Total Caregiver Burden (extroversion, agreeableness, conscientiousness) while neuroticisim has significant positive correlation with caregiving burden. Openness was not associated with caregiving burden (Table 3).

Majority of the coping strategies have positive correlation with caregiving burdern (i.e. self-distraction, active coping, denial, instrumental support, venting, positive reframing, planning and acceptance) while substance use, emotion support behaviour disengagement, humour, religion and self-blame were not significantly correlated with Total Caregiver Burden (Table 3).

### The mediating role of caregivers personality styles in the relationship between BPSD and caregiver burden

Multiple regressions were used to investigate if personality styles and coping strategies mediated the relationship between BPSD and Total Caregiver Burden. Each subscale of personality styles and coping strategies were analyzed separately to determine the mediation effect. Subscales that were not significantly correlated in Table 3 were excluded from further mediation analysis.

Table 4 below presents the results of 4-steps multiple mediation analyses of BPSD and caregiver burden with personality styles (extroversion, agreeableness, conscientiousness, and neuroticism) as the proposed mediator. In step 1 (path c) of mediation model, the regression of the total effect of BPSD Severity on Total Caregiver burden was significant (b = .5382, t (200) = 4.4311, *p* = <.05). Step 2 (path a) signified that the regression of the BPSD severity on the mediator (Extroversion) was not significant (b = −.0113, t (200) = −.2649, *p* = .7913), thus, no further mediation analysis was conducted as the *p*-value was greater than .05 which breached the requirement that all paths need to be significant. This indicates that Extroversion does not mediate the relationship between BPSD and Total Caregiver Burden. Similarly, Agreeableness and Neuroticism do not mediate the relationship between BPSD and caregiver burden. However, for Conscientiousness, step 1 (path c) of the mediation model, the regression of the total effect of BPSD Severity on Total Caregiver burden was significant (b = .5382, t (200) = 4.4311, *p* = <.001). Step 2 (path a) signified that the regression of the BPSD severity on mediator (Conscientiousness) was still significant (b = −.1552, t (200) = − 3.3781, *p* = .0009). Step 3 (path b) showed that the mediator (Conscientiousness), controlling for BPSD severity was significant (b = −.9172, t (199) = − 5.2159, *p* < .05). Step 4 (path c’) revealed that by controlling for the mediator (Conscientiousness), BPSD Severity was still significant (b = .3959, t (199) = 3.3717, *p* = .0009). Thus, this pattern indicates that Conscientiousness only partially mediate the relationship between BPSD severity and total caregiver burden.

**Table 4:** Multiple regression of BPSD and caregiver burden with personality style as the proposed mediator

	Paths	Coefficient	t	Sig.
Extroversion	**Step 2-** Path a: IV to mediator	.011	.2649	.7913
	**Step 3-** Path b: Direct effect of Mediators on DV	−.570	−2.889	.0043**
	**Step 1-** Path c: Total Effect of IV on DV	.538	4.431	.000***
	**Step 4-** Path c’: Direct Effect of IV on DV	.545	4.565	.000***
Agreeableness	**Step 2-** Path a: IV to mediator	−.079	−1.643	.1019
	**Step 3-** Path b: Direct effect of Mediators on DV	−.819	−4.839	.0000***
	**Step 1-** Path c: Total Effect of IV on DV	.538	4.431	.0000***
	**Step 4-** Path c’: Direct Effect of IV on DV via agreeableness	.474	4.083	.0001***
Conscientiousness	**Step 2-** Path a: IV to mediator	−.155	−3.378	.0009***
	**Step 3-** Path b: Direct effect of Mediator on DV	−.917	−5.215	.0000***
	**Step 1-** Path c: Total Effect of IV on DV	.538	4.431	.0000***
	**Step 4-** Path c’: Direct Effect of IV on DV	.396	3.372	.0009***
Neuroticism	**Step 2-** Path a: IV to mediator	−.536	−1.016	.3111
	**Step 3-** Path b: Direct effect of Mediators on DV	.734	4.743	.0000***
	**Step 1-** Path c: Total Effect of IV on DV	.538	4.431	.0000***
	**Step 4-** Path c’: Direct Effect of IV on DV	.578	4.991	.0000***

** *p* < .01

*** *p* < .001

### The mediating role of caregivers coping strategies in the relationship between BPSD and caregiver burden

Table 5 below presents the results of 4-steps multiple mediation analyses of BPSD and caregiver burden with coping strategies as the proposed mediator (self-distraction, active coping, denial, instrumental support, positive reframing, planning and acceptance) as the proposed mediator. In Step 1 of the mediation model (path c), the regression of the total effect of BPSD Severity on Total Caregiver burden was significant (b = .5382, t (200) = 4.4311, *p* < .05). Step 2 (path a) showed that the regression of the BPSD severity on the mediator (self-distraction) was also significant (b = .0587, t (200) = 3.4268, *p* = .007). Step 3 (path b) of the mediation process showed that the mediator (self-distraction), controlling for BPSD Severity, was significant (b = 3.1148, t (199) = 6.9002, *p* < .001). Step 4 (path c’) of the analyses revealed that, controlling for the mediator (self-distraction), BPSD severity was also significant (b = .3553, t (199) = 3.1571, *p* = .0018). Therefore, it indicates that self-distraction only partially mediate the relationship between BPSD severity and total caregiver burden. The same result was shown by active coping, planning and acceptance (Table 5). Denial, instrumental support, venting, and positive reframing coping strategies were not mediator between relationship BPSD Severity and Total Caregiver Burden.

**Table 5:** Multiple regression of BPSD and caregiver burden with coping strategies as proposed mediator

	Paths	Coefficient	t	Sig.
Self-distraction	**Step 2-** Path a: IV to mediator	.059	3.427	.0007***
	**Step 3-** Path b: Direct effect of Mediators on DV	3.115	6.900	.0000***
	**Step 1-** Path c: Total Effect of IV on DV	.538	4.431	.0000***
	**Step 4-** Path c’: Direct Effect of IV on DV	.355	3.157	.0018***
Active Coping	**Step 2-** Path a: IV to mediator	.065	4.593	.0000***
	**Step 3-** Path b: Direct effect of Mediators on DV	2.209	3.735	.0002***
	**Step 1-** Path c: Total Effect of IV on DV	.538	4.431	.0000***
	**Step 4-** Path c’: Direct Effect of IV on DV	.395	3.195	.0016***
Denial	**Step 2**- Path a: IV to mediator	.022	1.656	.0992
	**Step 3-** Path b: Direct effect of Mediators on DV	1.146	1.812	.0714
	**Step 1-** Path c: Total Effect of IV on DV	.538	4.431	.0000***
	**Step 4-** Path c’: Direct Effect of IV on DV	.513	4.215	.0000***
Instrumental support	**Step 2-** Path a: IV to mediator	.020	1.489	.1382
	**Step 3-** Path b: Direct effect of Mediators on DV	1.945	3.109	.0022***
	**Step 1-** Path c: Total Effect of IV on DV	.538	4.431	.0000***
	**Step 4-** Path c’: Direct Effect of IV on DV	.499	4.176	.0000***
Venting	**Step 2-** Path a: IV to mediator	.023	1.636	.1034
	**Step 3-** Path b: Direct effect of Mediators on DV	2.957	5.129	.0000***
	**Step 1-** Path c: Total Effect of IV on DV	.538	4.431	.0000***
	**Step 4-** Path c’: Direct Effect of IV on DV	.470	4.083	.0001***
Positive reframing	**Step 2-** Path a: IV to mediator	.028	1.791	.0748
	**Step 3-** Path b: Direct effect of Mediators on DV	1.778	3.323	.0011***
	**Step 1-** Path c: Total Effect of IV on DV	.538	4.431	.0000***
	**Step 4-** Path c’: Direct Effect of IV on DV	.488	4.087	.0001***
Planning	**Step 2-** Path a: IV to mediator	.077	6.045	.0000***
	**Step 3-** Path b: Direct effect of Mediators on DV	3.008	4.660	.0000***
	**Step 1-** Path c: Total Effect of IV on DV	.538	4.431	.0000***
	**Step 4-** Path c’: Direct Effect of IV on DV	.308	2.449	.0152*
Acceptance	**Step 2-** Path a: IV to mediator	.079	4.669	.0000***
	**Step 3-** Path b: Direct effect of Mediators on DV	2.644	5.588	.0000***
	**Step 1-** Path c: Total Effect of IV on DV	.538	4.431	.0000***
	**Step 4-** Path c’: Direct Effect of IV on DV	.329	2.764	.0062***

** *p* < .01

*** *p* < .001

## Discussion

The frequency of BPSD among patients with dementia in this study indicated that irritability was the most frequent type of BPSD exhibited by PWD (84.2%), followed by apathy (80.7%) and agitation (77.2%) as reported by caregivers (Table 2). Caregivers also implied that the least reported types of BPSD were elation (32.7%), followed by motor disturbance (57.5%) and appetite (59.4%)(Table 2). A previous study conducted in Malaysia showed that apathy was the most prevalent (83.2%), followed by agitation (60%) and sleep disturbance (53.8%) [7]. Past studies have concluded that neuropsychiatric symptoms in PWD are heterogeneous and essentially unpredictable in the presentation of emotional experience, thought content, perception and motor function which may explain the vast findings of research on prevalence of BPSD. However, despite the general discrepancy, there has been previous research where similar findings can be reported such as a study conducted by Mukherjee et al. [49] which highlighted that apathy/indifference was the most frequent (72.9%), followed by agitation/aggression (68.2%), and irritability/lability (59.8%). Aberrant motor behavior (31.8%), delusions (29%), and hallucinations (23.4%) were less frequent, while disinhibition (13.1%) and elation/euphoria (9.3%) were rare.

The pattern of previous findings is almost similar to the current research in which the three most frequent types of BPSD were reported accordingly while the least frequent types of BPSD were also highlighted in similar pattern with motor disturbance and elation being one of the least frequently reported BPSD. However, the results from other studies were inconsistent depending on different types of BPSD, the number of BPSD studied, environmental parameters and instrument used [5, 6, 17, 50, 51].

The pattern in which apathy is found to be one of the most common type of BPSD across several studies could be due to the presentation of the syndrome with reduced initiation and motivation, decreased social engagement, emotional indifference that could be misidentified with depression. PWD is rarely able to express pathological feelings of sadness, unhappiness, and preoccupation with depressing topics, hopeless (strongly associated with suicidal ideation) and loss of self-esteem [52]. As dementia progresses, other BPSD may predominate. Increased cognitive impairment was associated with more activity disturbances, hallucinations, agitation and sleep disturbances; however, delusions, affective disturbances, anxieties and phobias improved with worsening of the cognitive status [50]. Psychosis occurred more frequently with declining cognition and anxiety; depression were more common in younger patients [8].

In this study, the context of caregiving is primarily within vicinity of home-based settings which means that caregivers have more time providing care to PWD compared to when they are being institutionalized or sent to nursing home where most of past research have been focused on, thus, differences in reports as they varied in duration of their observation and presentation.

Table 3 indicated that Total BPSD severity score was significantly correlated with Total Caregiver Burden (r = .199, *p* < .01) which imply that the higher the severity of BPSD, the higher the level of caregiver burden. Of all the types of BPSD, delusion, agitation, irritability and nighttime behavior, hallucination, depression, apathy, disinhibition and motor disturbance were significantly correlated to total caregiver burden except for anxiety, elation and appetite.

Behavioral and psychological symptoms of dementia (BPSD) are very common and are significant symptoms of the illness, contributing most to caregiver burden and often resulting in premature institutionalization of the person with dementia. According to International Psychogeriatric Association [53], among the most intrusive and difficult BPSD types to cope comprise of delusions, hallucinations, depression, and anxiety. Past studies have indicated similar findings in which delusion and agitation were significantly associated with caregiver burden; thus, treatments for such BPSD may reduce the associated burden [54].

Delusions were seen in 14% of patients, were often seen early in the course of the disease, and were prominent and persistent. The presentation of delusion could instigate subsequent negative reactions by PWD that can be manifested in physical forms as a reciprocal response to caregivers. This is supported by studies that suggested that delusions are a risk factor for physical aggression. A study by Deutsch et al. [55] found that 43.5% of patients with a diagnosis of probable AD had delusions. Gilley et al. [56] also reported that the presence of delusions predicts the occurrence and frequency of physical aggression, with 80% of study participants who showed high rates of physical aggression (i.e. more than one episode per month) also having delusions. Symptoms like delusional thinking, activity disturbances and aggressiveness were more likely than other symptoms to be rated as troublesome to the caregiver.

Agitation has been found to correlate strongly with irritability, disinhibition, and delusions. Agitation and aggression are among the most troublesome BPSD symptoms for caregivers and, along with depression and psychosis, are leading predictors of institutionalization [57]. Sundowning is the occurrence and exacerbation of BPSD in the afternoon or evening. Agitation and sleep disturbances commonly accompany sundowning which increases the burden of care on caregivers, as it often occurs when the family members are at the lowest level.

In addition to psychotic symptoms and physical aggression, mood disturbance and disinhibition can also contribute greatly to caregiver burden. Current finding shows that depression (r = .157), apathy (r = .379) and disinhibition (r = .201) correlate significantly with caregiver burden. Depression may be especially challenging for caregivers to handle not only because of the difficulty it causes caregivers in dealing with the patients but also because of the negative impact it has on the patient’s quality of life. Anger/aggression (26%) and depression (17%) were the most frequently cited patient symptoms having impact on caregiver burden. Shaji et al. [17] reported that delusions, activity disturbances and aggression were perceived by caregivers to be more troublesome at times than memory deficits.

Current findings reported that there were a number of types of BPSD that were not significantly correlated to total caregiver burden which comprised of anxiety, elation and appetite. This could be explained by the fact that BPSD symptoms such as appetite is deemed less intrusive compared to delusion and agitation which includes physical violence. They cannot be easily dismissed by caregivers as they make them distressed, hence increase on caregiving burden. Disruptive behaviors are more disturbing partly because of the adverse impact on the emotional connection between the caregiver and the care-recipient and partly because they exacerbate difficulties in other domains (e.g., caring for activities of daily living) [58].

Although disinhibition (61.4%) and motor disturbance (57.5%) were found to be one of the least frequently reported type of BPSD, the presentation of respective symptoms has been shown to have strong correlation with caregiver burden which could mean that the influence of BPSD on caregiver burden is not directly related to how common or frequently the symptoms are but more of the underlying experiences of caregiving from the respective BPSD. Thus, it is to be underscored that the burden associated with BPSD is different for each symptom and does not always depend on frequency and severity of BPSD but could be extended to the nature of BPSD. These findings suggest that some symptoms, such as agitation/aggression and irritability/lability, as well as disinhibition and motor disturbance may affect the caregivers significantly, although their frequency and severity are low [54].

### Mediating effect of coping strategies and personality styles

Results reported conclude that majority of subscales in coping strategies mediate the relationships between BPSD and caregiver burden. Of coping strategies, self-distraction, active coping, planning and acceptance were found to mediate the relation between BPSD and caregiver burden whereas for personality styles, conscientiousness was the only subscale found to mediate the relationship. However, the mediation of endorsed coping strategies and personality style were all found to have partial mediation effect to the relationship.

In general, a given variable may be said to function as a mediator to the extent that it accounts for the relation between the predictor and the criterion. Mediators explain how external physical events take on internal psychological significance. The research model used in this study diagrams the mediating process by which the stressor precedes and influences the mediator and therefore affects the outcome [48].

The Multidimensional Stress-Process Model (SPM) posited how multiple stress factors contribute to negative outcomes for caregivers which was categorized into four types of variables that affect the well-being of caregivers: contextual variables, primary objective stressors, secondary stressors, and modulating variables. Based on the model, the most prominent primary stressors investigated are BPSD whereas coping strategies have a modulating function of different individual responses to the same care situation [59]. In a recent study that investigated the model also found that personality is one of the internal mediators to the relationship between primary stressor and outcome [60]; however, there has been lack of studies that have looked into the personality traits that have mediation effect in relation to BPSD and caregiving.

In the current research, it was reported that most coping strategies were found to mediate the relationship between BPSD and caregiver burden. This is supported by Lazarus [61] who argues that coping is a powerful mediator of the emotional outcome resulting from a stressful environmental transaction. Studies conducted by Folkman and Lazarus [62] highlighted that emotional state of the individual during the stressful encounter changed either positively or negatively based upon the type of coping strategy that was used. Although current research is not investigating on the direction of association of specific coping strategies and personality style; however, it does reveal that with the inclusion of self-distraction, active coping, planning and acceptance as well as personality characteristic of conscientiousness, they signified to be partially accounted for the relationship between BPSD and caregiver burden.

According to Lazarus and Folkman [63], there is no clear guidelines on whether coping effort is deemed successful but instead is more dependent upon the caregivers’ appraisal if the transaction with the environment was adequately resolved. This judgment is made based on the individual’s personality characteristics, values, beliefs, and expectations related to the different factors involved in the encounter. Coping process and strategies selected are not inherently good or bad.

Based on the current findings, it reveals that most of the highlighted mediators are problem-focused strategies which include defining the problem, generating alternative solutions, weighing the alternatives in terms of their costs and benefits, choosing among them, and acting [63]. Problem-focused coping is used when the individual makes a change with his/her relationship with the perceived stressor, such as working to fix a discrepancy between one’s current situation and what one wants. This is supported by a study conducted by Borden [64] which indicated that problem-focused coping directly contributed to caregivers having positive focus and therefore mediating psychological well-being.

This may suggest that strategies that are more emphasized on resolving stressor, which in this case is BPSD, influence the interaction with outcome. This is supported by Essex, Seltzer and Krauss [65] who found that greater use of problem-focused coping strategies and less use of emotion-focused coping techniques buffered the negative impact of stress on caregivers’ well-being. Similarly, in a study conducted by Miller et al. [66], it revealed that emotion-focused coping was significantly related to increased psychological distress in caregivers whereas use of problem-focused coping was tied to decreased distress. However, given that Williamson and Schulz [67] found emotion-focused coping more effective and problem-focused coping ineffective for AD caregivers indicates that coping style requires further study. This brief review of the literature demonstrates that the AD studies examining mediating models are at best inconsistent, and at times conflicting.

There is indeed increasing evidence that individual differences in personality may affect how carers experience and respond to the caregiving role. This is supported by Kobasa and Puccetti [68] who stated that personal characteristics affect health outcomes through coping strategies. Personality characteristics affect the processes that individuals use to appraise stressful events and predispose them to cope in certain ways when they confront these events [31].

Previous studies have noted that personality variables are consistently associated and predictive of a range of outcomes in dementia carers and that they are of important predictive value in terms of outcomes for people with dementia. Results in the current finding indicated that out of all the five-taxonomy personality traits, conscientiousness was found to have partial mediation effect on the relationship between BPSD and caregiver burden.

This can be explained by the fact individuals who score high on this trait are self-disciplined and organized, which is linked to greater health-promoting behaviors which would result in better subjective and objective health [69]. Highly conscientious individuals also report a sense of competence and confidence, and this may partially account for their apparently better mental health [70]. Thus, due to the nature of the personality, it provides a strong proclivity for caregivers to process the stress and implement strategies that are effective in reducing the stressor which in return would lessen the caregiver burden.

According to the results, it can be seen that there is a pattern between the mediator coping strategies that are more problem-focused which share similar characteristics as being conscientiousness whereby both factors consistently emphasize on organization, efficiency, orderly and structure. The shared components are based on managing the intensity and complexity of primary stressor which would in turn influence the level of caregiver burden. Although there is no significance in association between being conscientiousness and the endorsed coping strategies to show how personality trait is a predisposition to coping responses; however, the shared similarities may provide substantial interpretation on the relationship between BPSD and the outcome of caregiver burden which can be explained through responses and characteristics that are systematic, organized and planned.

Thus, this imply that caregiver burden is not so much from the frequency of the behavior, but it could be based on the nature of the types BPSD that are appraised by caregivers who manage these PWD at home.

There were few limitations in this study. Firstly, the lack of consideration of the duration of caregiving and how it may play a role in the burden of care. Longer duration of caregiving and more experienced caregivers may not feel much caregiver burden if they have acquired suitable coping strategies that have been effective in managing their stress level over time. Secondly, there is lack of specificity on different stages of dementia and how it can influence of emergence of BPSD. Caregivers of PWD were not provided with specific details of severity levels unless requested. Thus with the limited information of severity level of dementia, the presentation of BPSD is not exclusively delineated according to according to the presentation of the disease but more of how BPSD in general is associated with caregiver burden.

Future research should take into consideration the information on PWD’s disease progression to get a more refined understanding on presentation of BPSD to the outcome of caregiver burden. Future studies also should look into the mediating role of personality styles that can explain the relationship between stressor and caregiver burden.

The study provides implication on a fundamental understanding that the frequency of BPSD is not necessarily associated with caregivers’ burden level but more into the nature of the BPSD. Based on the association of BPSD to caregiver burden, it provides inferences that delusion, agitation, irritability and nighttime behavior are among the main behaviors to intervene on PWD first as they demonstrated to be highly correlated to caregivers’ burden level. Another implication of the study is to prevent caregiver burnout by suggesting that it is crucial to increase coping skills that are more problem-focused and action-oriented that share components of being systematic, thorough and organized as they have shown to mediate the relationship to the outcome.

## Conclusion

The findings of the current study provide a greater insight on frequency of BPSD types in Malaysia, correlates of BPSD on caregiver burden as well as the mediation effect of coping strategies and personality styles. It was revealed that the highest most frequently reported type of BPSD exhibited by PWD was irritability, followed by apathy and agitation. In regard to correlations between BPSD and caregiver burden, it was revealed that the highly correlated BPSD to caregiver burden are mostly those that are most intrusive for the caregivers. Coping strategies such a self-distraction, active coping, planning and acceptance as well as conscientiousness personality trait were shown to mediate the relationship between BPSD and caregiver burden. The study implies that it is crucial to include information on PWD’s disease progression in order to tie in the emergence of BPSD to the severity level to get a more refined understanding on presentation of BPSD to the outcome of caregiver burden. Attention also should be given on how personal characteristics can actually explain the relationship between stressor and caregiver burden. As current finding revealed that only one personality trait demonstrated mediating effect, it would be suggested for future research to build on the understanding by investigating the types of personality traits and their mediation.

## Abbreviations

AD: Alzheimer’s disease
BFI: Big-Five Inventory
BPSD: Behavioral and Psychological Symptoms of Dementia
NPI-Q: Neuropsychiatric Inventory-Questionnaire
PWD: Patients with dementia
SPM: Stress-Process Model
SPSS: Statistical Package of Social Sciences
ZBI: Zarit Burden Interview
